# Efficacy and safety of full-endoscopic sacroiliac joint denervation for the treatment of chronic low back pain: a systematic review

**DOI:** 10.1007/s00701-026-06807-5

**Published:** 2026-02-25

**Authors:** Dia R. Halalmeh, Yusuf-Zain Ansari, Arwa Jader, Rahul Kumar, Amy Herrera, Saqib Hasan

**Affiliations:** 1 Department of Neurosurgery, University of Iowa Hospitals and Clinics, Iowa City, IA USA; 2 College of Science and Technology, Temple University, 1801 N Broad St, Philadelphia, PA 19122 USA; 3 Department of Neurosurgery, University of Kufa, Kufa, Iraq; 4 University of Miami Miller School of Medicine, Miami, FL USA; 5 Golden State Orthopedics and Spine, Oakland, CA USA

**Keywords:** Chronic low back pain, Full-endoscopic denervation, Minimally invasive spine surgery, Radiofrequency neurotomy, Sacroiliac joint dysfunction

## Abstract

**Background:**

Full-endoscopic sacroiliac joint denervation (FE-SJD) is a novel minimally invasive technique that increases the precision of targeting the nerve roots responsible for chronic low back pain (CLBP). The purpose of this study is to evaluate the safety and efficacy of FE-SJD for the management of CLBP attributable to sacroiliac joint (SIJ) dysfunction.

**Methods:**

A systematic search of PubMed/MEDLINE, Cochrane Library, and Google Scholar using the Preferred Reporting Items for Systematic Reviews and Meta-Analyses (PRISMA) statement was conducted to collect studies assessing the effectiveness and safety of FE-SJD in the treatment of CLBP of SIJ origin.

**Results:**

A total of 6 studies encompassing 169 patients were included. The weighted age of patients was 59.95 ± 12.07 years with 102 (60.36%) females. The weighted mean of BMI was 26.82 ± 4.62. All patients presented with SIJ-attributable CLBP, with an average duration of 55.0 ± 28.0 months. The most common treated level was L4-5 with a mean operation time of 44.0 ± 11.2 min. The preoperative Visual Analog Scale (VAS) and Oswestry Disability Index (ODI) weighted mean were 6.675 ± 1.294 and 34.4 ± 6.99 which significantly improved to 2.46 ± 1.65 and 12.3 ± 5.47 at the latest follow-up, respectively (*P* < .001). The mean follow-up period was 18 ± 9.75 months. There were no complications.

**Conclusion:**

The available evidence suggests that FE-SJD is associated with favorable clinical outcomes and a low complication rate in the treatment of chronic low back pain attributable to sacroiliac joint dysfunction. However, the current literature is limited by small sample sizes and predominantly non-comparative study designs.

## Introduction

Low back pain is a pervasive and debilitating health issue, affecting an estimated 18% to 80% of the population globally and contributing to over $50 billion in annual healthcare costs in the United States alone [3, 9, 10]. Although many patients recover, a significant proportion develop chronic low back pain (CLBP), which places an ongoing burden on healthcare systems due to prolonged treatments, hospital stays, and associated costs [13]. Among the various etiologies of CLBP, dysfunction of the sacroiliac joint (SIJ) is particularly prominent, with SIJ dysfunction accounting for 15% to 30% of LBP cases [4, 5].

Historically, the surgical management of CLBP has evolved significantly. The initial novel SIJ arthrodesis performed by Smith-Petersen and Rogers in 1926 laid the groundwork for a century-long trajectory of surgical development [15]. Early surgical approaches involved the fusion of the joint to stabilize and reduce pain. These procedures, while effective for some, were often associated with long recovery times and significant postoperative complications [14]. As a result, there was a period when surgical intervention was less favored, and conservative treatments were more commonly pursued [18]. However, with advancements in technology and techniques, interest in surgical solutions for SIJ dysfunction resurfaced. Minimally invasive surgical techniques have revolutionized the field by offering less invasive options with reduced recovery times and lower complication rates.

In recent decades, the development of various denervation techniques has further shifted the surgical management of CLBP away from fusion. Denervation, including radiofrequency ablation (RFA), has shown promising results in improving pain relief, patient satisfaction, and functional outcomes [1]​. Full-endoscopic sacroiliac joint denervation (FE-SJD), a novel minimally invasive technique, has emerged as a promising approach for targeting the nerve roots responsible for CLBP with greater precision compared to traditional methods. Despite its potential, data on the long-term efficacy and safety of FE-SJD remain limited, necessitating further research to establish its role as a viable alternative to conventional treatments.

This systematic review aims to elucidate the safety and efficacy of FE-SJD for the management of CLBP by analyzing pooled data from multiple studies to provide a comprehensive assessment of this technique, identify gaps in the current literature, and offer insights that may guide future research and clinical practices in the treatment of CLBP.

## Methods and materials

### Literature search

A systematic review was performed in accordance with the Preferred Reporting Items for Systematic Reviews and Meta-Analyses (PRISMA) statement. In August 2024, systematic database investigations were undertaken encompassing PubMed/MEDLINE, Cochrane Library, and Google Scholar. Additional sources included reference lists of relevant reviews and original articles to ensure comprehensive coverage. The date of the last search was November 2024.

The devised search algorithm utilized Boolean conjunctions ("AND", "OR") incorporating the following keywords: ((Endoscopic rhizotomy OR Endoscopic radiofrequency ablation OR Joint denervation) AND (Sacroiliac joint OR Chronic low back pain)). Post-search, all acquired studies were imported into RAYYAN for deduplication and blinded screening. The review was not registered in PROSPERO.

### Study selection

Eligibility criteria were defined prior to the literature search. Studies were included if they investigated the efficacy and safety of full-endoscopic techniques—uniportal or biportal—for treating SIJ attributable CLBP. Only studies presenting original clinical data were considered.

We excluded studies that investigated endoscopic approaches for indications other than SIJ pain, such as facet-mediated pain, lumbar discectomy, foraminal decompression, or debridement. Studies that did not provide relevant outcome data for patients with CLBP treated via endoscopic denervation were also excluded. Additional exclusion criteria encompassed non-English language publications; conference abstracts, book chapters, letters, and editorials; radiologic or anatomical studies without clinical data; and preclinical research involving animals or cadavers. While review articles were not eligible for inclusion, their reference lists were systematically screened to identify potentially relevant original studies.

Screening was performed by two independent reviewers (A.J., Y.A.), who evaluated all titles/abstracts and subsequently full-texts. Discrepancies were resolved by a third reviewer (D.H.). Automation tools (RAYYAN) assisted in deduplication but not eligibility determination. Following the pre-established guidelines, relevant articles were integrated, and their reference lists were examined for additional pertinent studies.

### Data extraction

Reviewers Y.A. and A.J. conducted the initial data extraction, with all entries independently verified for accuracy by reviewer D.H. Missing data were not reported in the original studies. Extracted variables included study author, year of publication, country of origin, study design, sample size, age, sex distribution, BMI, presenting symptoms, symptom duration (in months), preoperative Visual Analog Scale (VAS) and Oswestry Disability Index (ODI) scores, diagnosis, surgical details (approach, laterality, treated branch and vertebral level, anesthesia status), operative time, estimated blood loss, decompression adequacy (complete or partial), and intraoperative complications. Postoperative variables included complications, VAS and ODI scores, conversion to open surgery, reoperation, and follow-up duration.

### Data synthesis and statistical analyses

The primary outcome was centered on delineating the safety and efficacy of FE-SJD of patients with CLBP. Descriptive analyses were conducted using Jamovi (version 2.4.1.0). Continuous variables are reported as mean ± standard deviation (SD) for normally distributed data and as median with interquartile range (IQR) for non-normally distributed data. Categorical variables are summarized using frequencies and percentages.

For the meta-analysis, pooled mean differences and corresponding 95% confidence intervals (CIs) were calculated using the DerSimonian–Laird method. Changes in VAS and ODI scores from preoperative to postoperative assessments were analyzed. Statistical heterogeneity was assessed using the Higgins I^2^ statistic. A random-effects model was applied when I^2^ ≥ 40%, and a fixed-effects model was used when I^2^ < 40%, following Cochrane guidelines [8]. All meta-analyses were performed using OpenMeta[Analyst]. Statistical significance was defined as a 95% CI excluding the null value and a *P*-value < 0.05.

### Risk of bias assessment

We performed a risk-of-bias assessment using the Risk Of Bias In Non-randomized Studies – of Interventions (ROBINS-I) tool. Reviewers A.J. and Y.Z.A. independently evaluated each study across the ROBINS-I domains, including bias due to confounding, selection of participants, classification of interventions, deviations from intended interventions, missing data, measurement of outcomes, and selection of reported results. Any discrepancies were resolved through discussion and consensus.

## Results

### Literature search and study selection

**Fig. 1:**
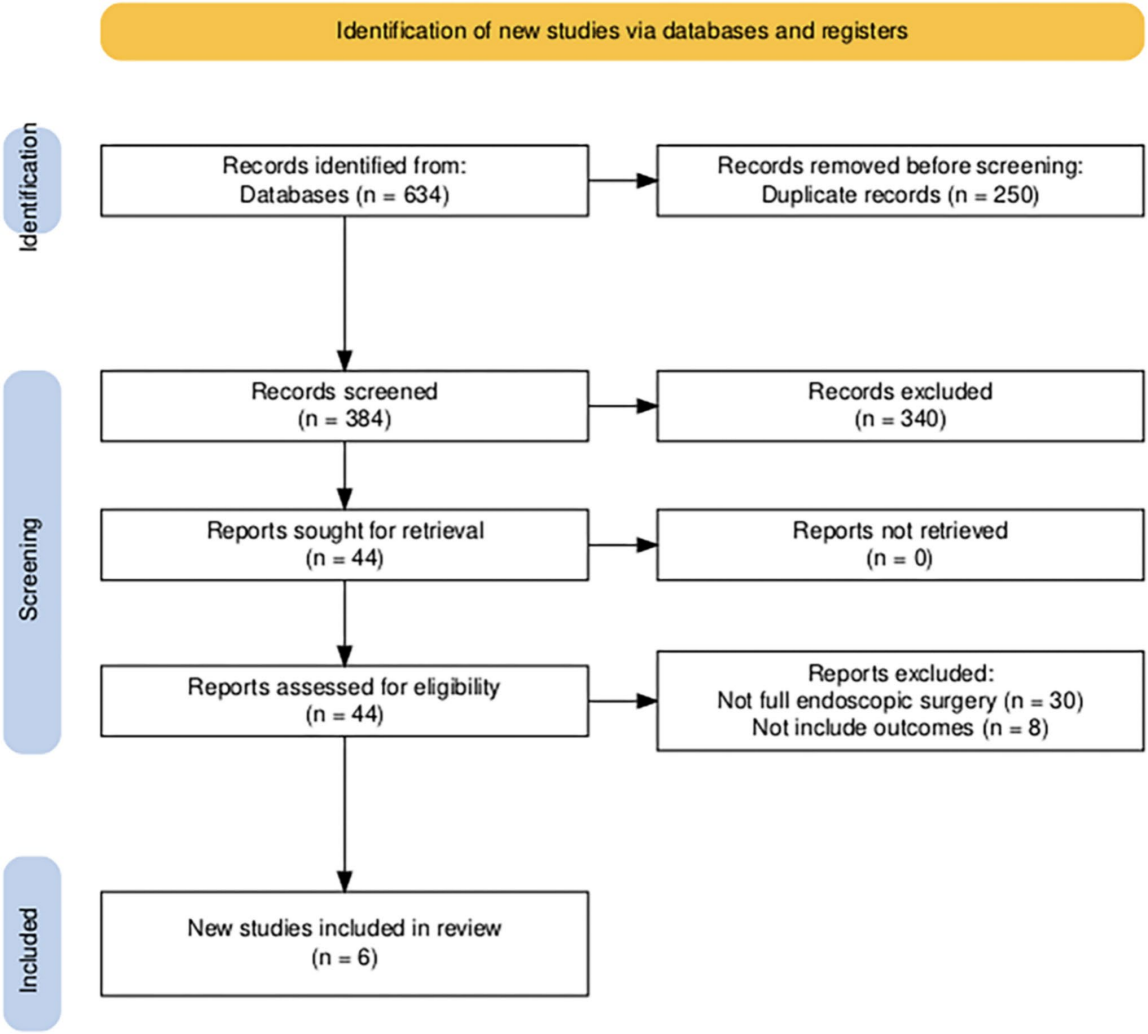
PRISMA flow diagram of study selection process

In this systematic review, we identified a total of 634 articles through database searches. After the removal of 250 duplicate records, 384 unique studies were screened based on titles and abstracts. Of these, 340 were excluded for failing to meet basic inclusion criteria. The remaining 44 full-text articles were retrieved and assessed for eligibility. Following full-text review, 38 articles were excluded. Six studies met all inclusion criteria and were included in the final analysis, encompassing a total of 169 patients. The study selection process is summarized in Fig. 1, in accordance with PRISMA guidelines. The characteristics and findings of these six studies are summarized in Table 1 [2, 3, 7, 11, 16, 17].

**Table 1:** Summary of included studies

Study	Study type	Region	Measured Outcomes
Chen et al.	Retrospective	Taiwan	VAS, ODI
Choi et al.	Retrospective	South Korea	VAS, ODI
Ibrahim et al.	Retrospective	Germany	VAS, ODI
Tseng et al.	Retrospective	Taiwan	VAS, ODI
Hasan et al.	Prospective	United States	VAS, ODI
Woiciechowsky et al.	Retrospective	Germany	VAS, Odom’s criteria

### Patient demographics, operative characteristics, and clinical outcomes

Across the included studies, a total of 169 patients underwent FE-SJD. The mean age was 59.95 ± 12.07, with 60.36% female. The weighted average BMI was 26.82 ± 4.62. 169 (100%) of the patients were diagnosed with sacroiliac joint attributable lower back pain, with an average length of symptoms before surgery of 55 ± 28 months. The mean follow-up period after surgery was 18.00 ± 9.75 months. A summary of these pooled demographic characteristics is displayed in Table 2.

**Table 2:** Patient demographics

Study	Sample size		% Male	Age	BMI	Follow up period
	Number	% Total				
Chen et al.	36	21.3%	36.1%	62.27 ± 2.37	25.68 ± 4.19	12
Choi et al.	17	10.1%	11.8%	61.90 ± 11.80	–	12
Ibrahim et al.	30	17.8%	36.7%	56.00	–	24
Tseng et al.	16	9.5%	–	–	–	12
Hasan et al.	47	27.8%	36.2%	59.40 ± 14.00	27.70 ± 4.80	24
Woiciechowsky et al.	23	13.6%	34.8%	59.60 ± 13.40	-	24
	169	100.0%	30.2%	59.95 ± 12.07	26.82 ± 4.62	18.00 ± 9.75

The most commonly targeted nerve branches were lateral branches from L5 to S3 with 75 (44.37%) and the mean operation duration was 44.00 ± 11.20 min. Preoperative assessments showed a mean VAS score of 6.675 ± 1.294 and a mean ODI score of 34.4 ± 6.99. Postoperatively, the mean VAS score significantly decreased to 2.46 ± 1.65, and the mean ODI score improved to 12.3 ± 5.47. The reductions in both VAS and ODI scores were statistically significant (*P* < 0.001), indicating a substantial improvement in pain and functional disability following the surgical intervention (Table 3).

**Table 3:** Operative and clinical outcome data

Study	Treated Branches	EBL	Operative Time (min)	Reoperation	VAS			ODI		
					Pre-op	Post-op	*P*-value	Pre-op	Post-op	*P*-value
Chen et al.	L5-S3	< 5 mL	39.08 ± 14.05	0	7.25 ± 1.66	1.14 ± 1.82	*P* < 0.001	20.80 ± 4.90	5.25 ± 6.54	*P* < 0.001
Choi et al.	L4-S3	–	26.60 ± 22.50	0	6.70 ± 1.41	3.10 ± 1.78	*P* < 0.005	22.20 ± 3.36	12.00 ± 4.69	*P* < 0.001
Ibrahim et al.	L4-S4	–	52	0	7.23 ± 1.55	2.82 ± 1.33	*P* < 0.001	44.80 ± 21.73	22.24 ± 19.09	*P* < 0.001
Tseng et al.	L5-S3	< 5 mL	–	0	7.25 ± 0.61	1.00 ± 0	*P* < 0.001	34.40 ± 6.99	12.30 ± 5.47	*P* < 0.001
Hasan et al.	L4-S3	–	57.10 ± 16.80	2	5.96 ± 0.33	3.16 ± 0.55	*P* < 0.001	39.82 ± 2.46	23.43 ± 4.39	*P* < 0.05
Woiciechowsky et al.	L5-S3	–	–	0	6.10 ± 0.82	3.20 ± 1.94	*P* < 0.001	–	–	–
	–	–	44.00 ± 11.20	2 (1.18%)	6.68 ± 1.29	2.46 ± 1.65	*P* < 0.001	34.4 ± 6.99	12.3 ± 5.47	*P* < 0.001

Two patients (1.18%) underwent reoperation throughout all the studies with both instances coming from the same paper [7]. Both of these patients were converted to open surgery for persistent symptoms despite complete endoscopic denervation. Notably, no complications were observed in any of the included studies.

### Meta-analysis of VAS and ODI outcomes

**Fig. 2:**
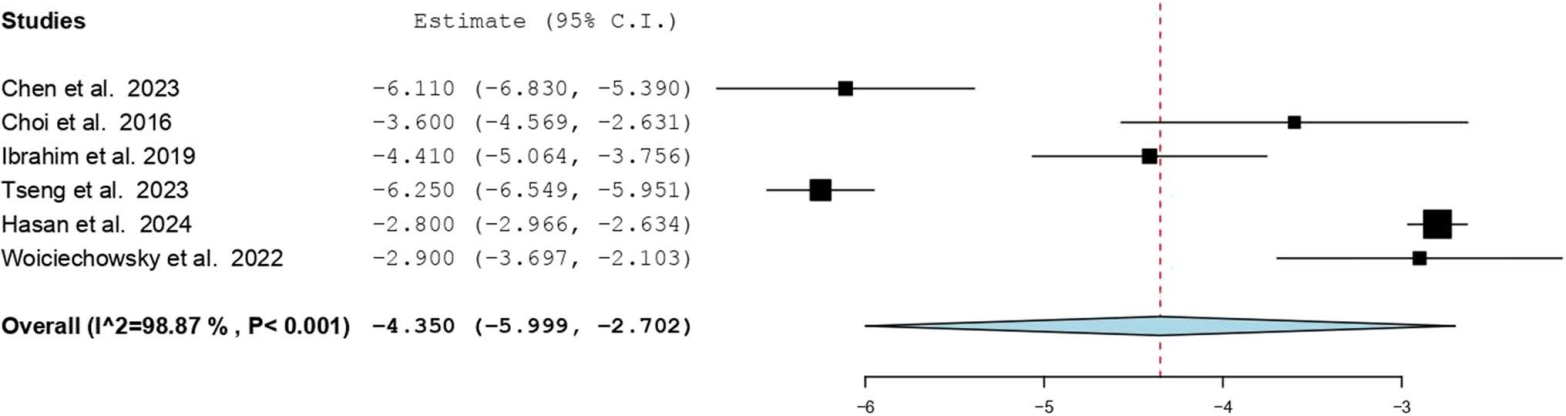
Forest plot of mean difference in VAS scores (Pre- vs. Postoperative)

A single-arm meta-analysis of six studies evaluated changes in VAS scores following FE-SJD [2, 3, 7, 11, 16, 17]. The pooled mean difference was –4.35 (95% CI: –5.999 to –2.702), indicating a statistically significant reduction in pain postoperatively. However, substantial heterogeneity was observed (I^2^ = 98.87%, *P* < 0.001), as depicted in Fig. 2, which displays the individual and pooled mean differences with corresponding 95% CIs.

**Fig. 3:**
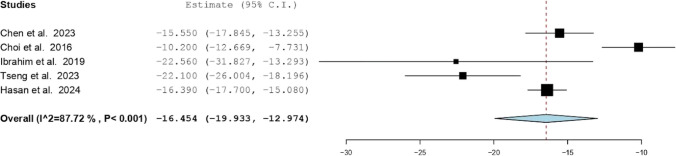
Forest plot of mean difference in ODI scores (Pre- vs. Postoperative)

Five studies were included in a single-arm meta-analysis for ODI scores [2, 3, 7, 11, 16, 17]. The pooled mean difference was –16.454 (95% CI: –19.933 to –12.974), again showing a statistically significant improvement in disability scores. Heterogeneity remained high (I^2^ = 87.72%, *P* < 0.001), as shown in Fig. 3.

To explore the observed heterogeneity, exploratory sensitivity analyses, subgroup analyses by vertebral level, and meta-regression based on gender were performed. However, given the small number of included studies, these analyses were inherently underpowered and did not meaningfully reduce heterogeneity.

### Risk of bias

**Fig. 4:**
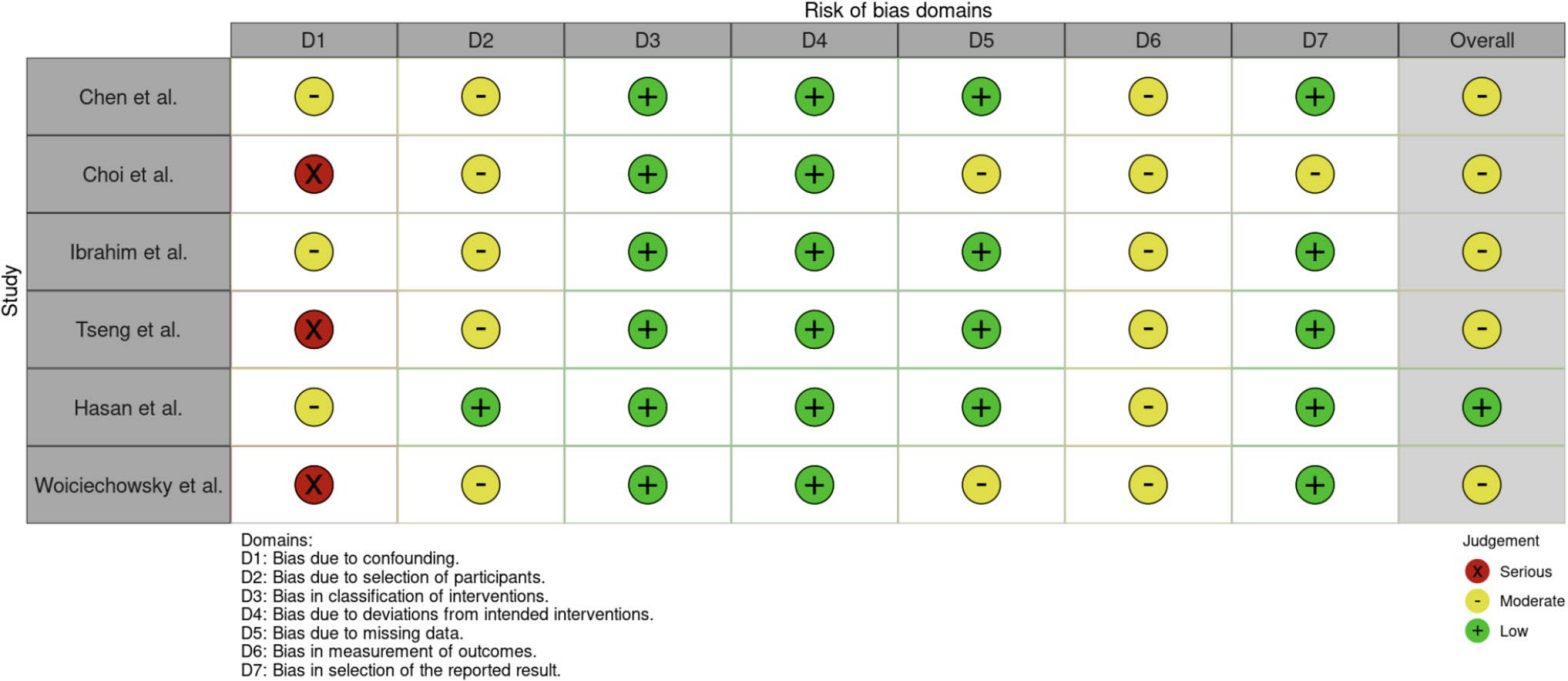
Traffic-light plot

The risk of bias of the included studies was assessed across seven domains using the ROBINS-I tool (Fig. 4). Most studies demonstrated low risk in the classification of interventions (D3) and deviations from intended interventions (D4), reflecting consistent application of the surgical techniques. Likewise, risk from missing data (D5) was generally low, with most studies achieving complete or near-complete follow-up.

However, common limitations were noted. Several studies were judged to be at serious risk of bias due to confounding (D1), as they lacked control groups or adjustment for important covariates such as prior spinal surgery or comorbid conditions. Risk of bias due to selection of participants (D2) was frequently moderate, reflecting retrospective enrollment and small sample sizes. Measurement of outcomes (D6) was also a source of moderate to serious bias, as all outcomes were self-reported without blinding. Finally, while most studies reported prespecified outcomes, selective reporting (D7) could not be fully excluded in some cases.

## Discussion

This systematic review of six studies encompassing 169 patients demonstrated that FE-SJD significantly improves pain and functional outcomes in CLBP secondary to SIJ dysfunction. Specifically, patients showed a substantial reduction in pain severity, measured by a decrease in VAS from a preoperative mean of 6.68 to 2.46 postoperatively (*P* < 0.001). Similarly, functional disability, assessed by the ODI, significantly improved from 34.4 ± 6.99 preoperatively to 12.3 ± 5.47 postoperatively (*P* < 0.001). No serious complications were reported across studies, and the reoperation rate was low (1.18%), indicating that FE-SJD is both safe and effective for SIJ-related CLBP.

One proposed technical advantage of FE-SJD is the ability to directly visualize the sacroiliac joint lateral branches during denervation. Conventional fluoroscopy-guided RFA techniques may be limited in their ability to consistently localize the lateral branches of L5–S3 due to anatomical variability and the depth of these nerve structures. In contrast, the endoscopic approach allows direct visualization of neural anatomy, which may facilitate more targeted ablation in select cases [7]. The enhanced visualization capabilities of the endoscopic approach allow for more precise targeting of deeply situated nerve branches, which are often shielded by soft tissue and ligamentous structures [6]. This direct visualization may reduce the risk of missing targeted nerves or inadvertently damaging nearby structures, likely contributing to the favorable outcomes and safety profile observed in the included studies.

Two patients across the included studies ultimately required conversion to open SIJ fusion. In both cases, symptoms persisted despite complete denervation, prompting subsequent open fusion. This finding raises the possibility of misclassification of pain etiology at the time of initial intervention. While FE-SJD may provide temporary symptomatic relief, it does not address underlying mechanical instability of the joint. Conversely, the diagnostic complexity of SIJ pain is further illustrated by reports of persistent or recurrent pain even after SIJ fusion. Lee et al. describes a 67-year-old patient who continued to experience significant SIJ-related pain despite prior bilateral open SIJ fusion [12]. The patient subsequently underwent RFA, which resulted in meaningful symptomatic improvement, highlighting that even after structural stabilization, nociceptive signaling from residual or adjacent neural structures may persist. Together, these observations emphasize that SIJ pain exists on a spectrum involving both mechanical instability and nociceptive sensitization.

### Limitations

Despite promising results, the present review has several limitations. The total sample size remains relatively small at 169 patients, which could affect the generalizability of the results. Additionally, the average follow-up duration was only 18 months, limiting insights into long-term outcomes, particularly important given the chronic and recurrent nature of SIJ pain.

Significant heterogeneity among the studies (I^2^ values of 98.87% for VAS and 87.72% for ODI) suggests variability in methodologies, surgical techniques, patient characteristics, and outcome measurements, complicating the interpretation of pooled results. While approach-related variables such as uniportal versus biportal techniques, differences in endoscopic visualization systems, and variation in follow-up duration are plausible effect modifiers in endoscopic SIJ denervation, the small number of studies and inconsistent stratified reporting precluded formal subgroup analysis of these variables. Not all studies clearly differentiated surgical approach in outcome reporting, and follow-up durations were variably reported without time-point–specific effect sizes suitable for meta-regression. As such, heterogeneity analyses were limited to variables that were consistently extractable across studies. Although exploratory subgroup and meta-regression analyses were conducted, the limited number of included studies substantially restricted statistical power and precluded more robust heterogeneity modeling or multivariable meta-regression.

Future research should focus on addressing these limitations. Larger, multicenter randomized controlled trials with standardized protocols are essential. Such studies would offer clearer evidence on patient selection, optimal surgical techniques, and long-term effectiveness. Comparative studies directly assessing endoscopic neurotomy against RFA and conservative treatments could further clarify its therapeutic value and cost-effectiveness. Finally, longer follow-up periods are necessary to determine the durability of pain relief and functional improvement provided by this innovative approach.

## Conclusion

FE-SJD represents a minimally invasive treatment option for CLBP secondary to SIJ dysfunction that demonstrates favorable short- to mid-term clinical outcomes and a low reported complication rate in the available literature. Analysis of the included studies indicated significant improvements in postoperative pain and functional disability outcomes. While endoscopic techniques allow direct visualization of relevant neural structures, comparative studies are required to determine whether this technical feature translates into superior clinical outcomes relative to conventional interventions.

## Data Availability

No datasets were generated or analysed during the current study.

## Abbreviations

RFA: Radiofrequency ablation
FE-SJD: Full-endoscopic sacroiliac joint denervation
CLBP: Chronic low back pain
SIJ: Sacroiliac Joint
VAS: Visual Analog Scale
ODI: Oswestry Disability Index
SD: Standard deviation
IQR: Interquartile range
CI: Confidence interval
PRISMA: Preferred Reporting Items for Systematic Reviews and Meta-Analyses
CI: Confidence interval

## Declarations

### Registration

Not registered in PROSPERO.
